# Publisher Correction: Global analysis of protein degradation in prion infected cells

**DOI:** 10.1038/s41598-020-69657-w

**Published:** 2020-07-28

**Authors:** Charles R. Hutti, Kevin A. Welle, Jennifer R. Hryhorenko, Sina Ghaemmaghami

**Affiliations:** 1 Department of Biology, University of Rochester, New York, 14627 USA; 2 University of Rochester Mass Spectrometry Resource Laboratory, New York, 14627 USA

Correction to: *Scientific Reports* 10.1038/s41598-020-67505-5, published online 01 July 2020

In Figure 4A and 4B, ‘ +QA/−QA’ should read as ‘−QA/+QA’. The correct Figure 4 appears below as Figure 1.

**Figure 1:**
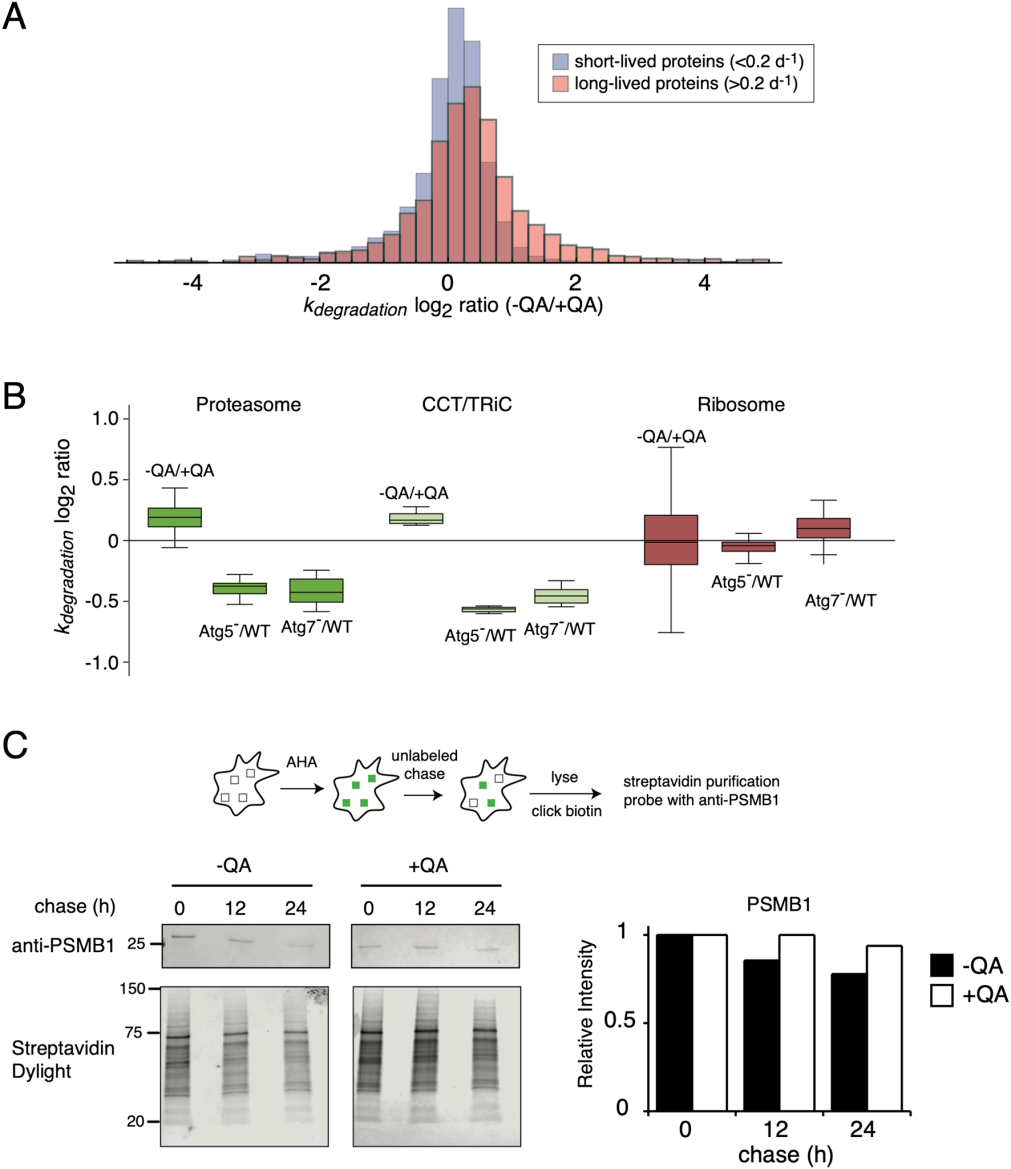
The degradation rates of long-lived proteins and autophagy substrates are increased in prion infected cells. (**A**) The log_2_ ratio of degradation rates between − QA and + QA samples for short-lived (k_degradation_ > 0.2 day^−1^) and long-lived (k_degradation_ < 0.2 day^−1^) proteins. (**B**) The effect of prion infection on k_degradation_ of two previously established substrates of basal autophagy (the proteasome and CCT/TRiC) and the ribosome, previously shown to be excluded from basal autophagy by Zhang et al. The change in degradation rates in autophagy-deficient cells (ATG5^−/−^ and ATG7^−/−^) compared to wildtype, measured by Zhang et al., is shown for comparison. (**C**) Analysis of PSMB1 degradation kinetics by AHA labeling. Cultures of − QA and + QA cells were labeled with AHA for 16 h and chased with unlabeled media for variable lengths of time. Labeled proteins were biotinylated by copper-mediated click chemistry and subsequently purified from total cell lysates using Streptavidin magnetic beads. Purified PSMB1 was detected through western blot analysis and compared to total purified protein. Relative intensities of labeled proteins were quantified as described in “Methods”.

