# Supplementary material for: Global analysis of protein degradation in prion infected cells
Source: Sci Rep. 2020 Jul 1;10:10800. doi: 10.1038/s41598-020-67505-5 (PMC7329860; doi:10.1038/s41598-020-67505-5)

SUPPLEMENTARY INFORMATION

Global analysis of protein degradation in prion infected cells

Charles R. Hutti[1], Kevin A. Welle[2], Jennifer R. Hryhorenko[2], Sina Ghaemmaghami[1,2*]

[1]Department of Biology, University of Rochester, NY, 14627, USA

[2]University of Rochester Mass Spectrometry Resource Laboratory, NY, 14627, USA

[*]Corresponding author: sina.ghaemmaghami@rochester.edu (S.G.)

Provided separately as Excel spreadsheets:

Table S1 – Peptide level search data

Table S2 – Protein level search data

Table S3 – Protein degradation rates

Table S4 – Optimized gradients for individual fractions during QExactive LC-MS/MS

Table S5 – Inclusion list for SILAC labeled PrP peptides generated using Skyline software program

Table S6 – MaxQuant software parameters for data analysis

Full-length western blots for the corresponding to the main figures in the text are provided on the following pages.

Figure 2 - A (left)

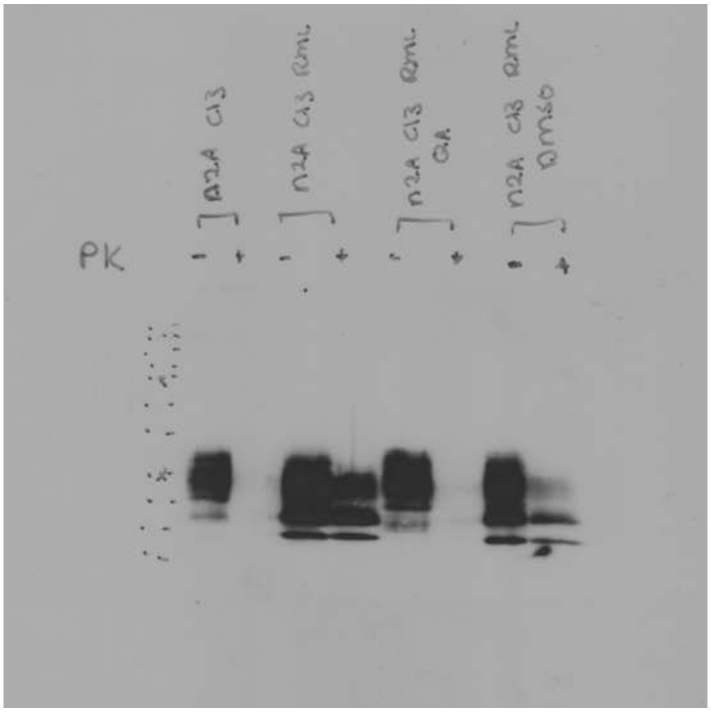

Figure 2 - A (right)

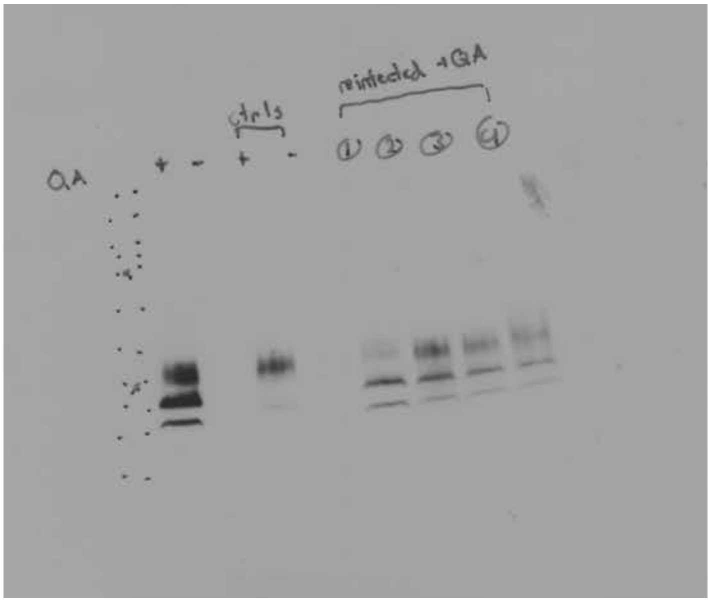

Figure 3 - D (left)

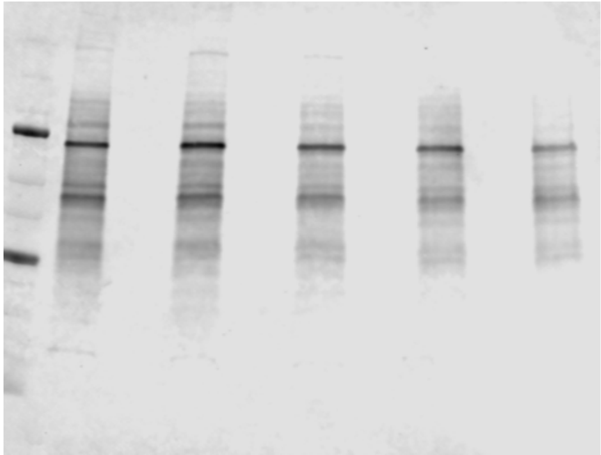

Figure 3 - D (right)

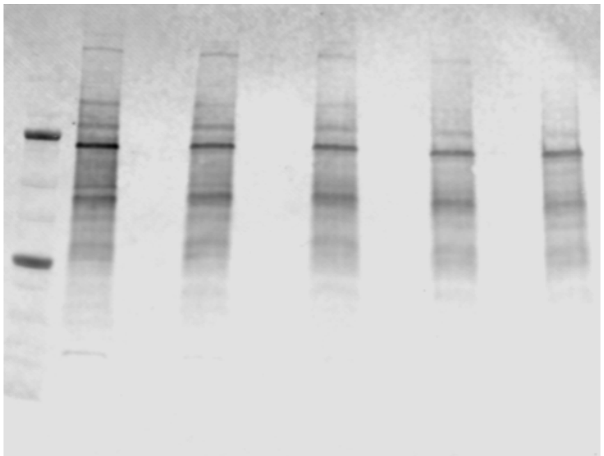

Figure 4 - C (upper left)

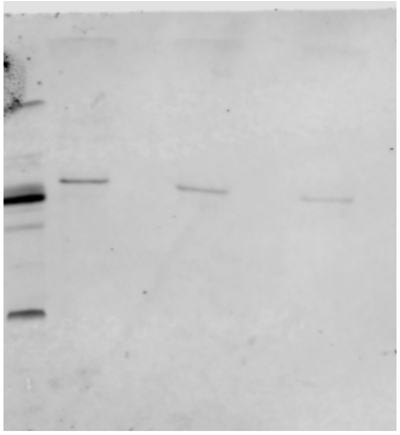

Figure 4 - C (upper right)

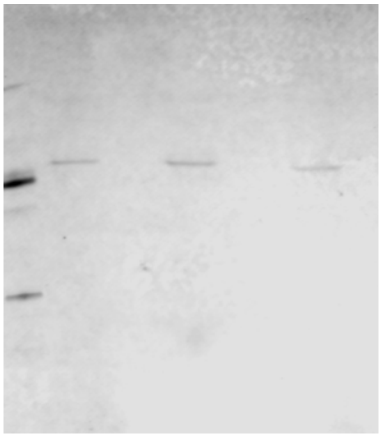

Figure 4 - C (lower left)

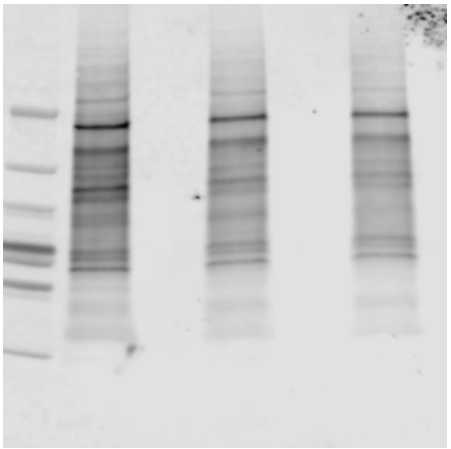

Figure 4 - C (lower right)

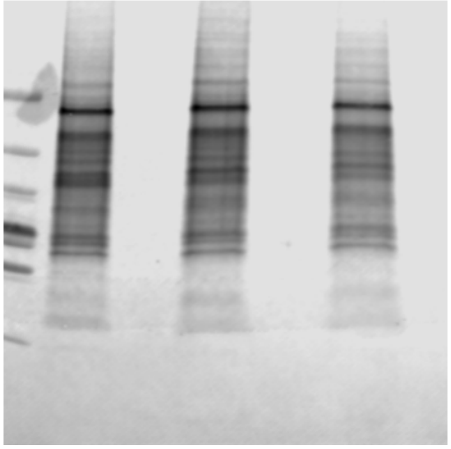

Figure 5 - A (cathepsin D)

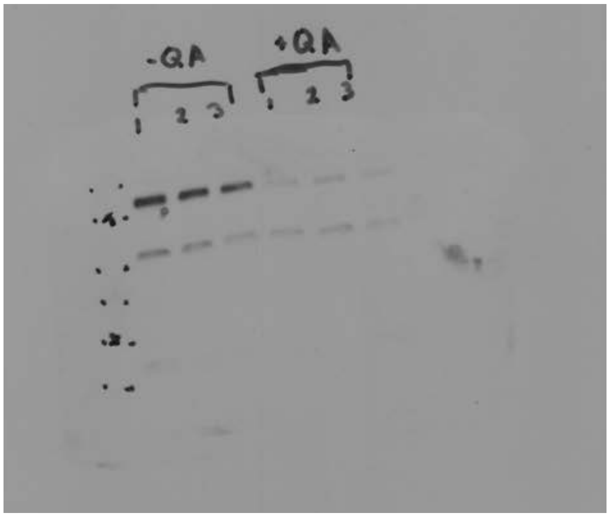

Figure 5 - A (cathepsin A)

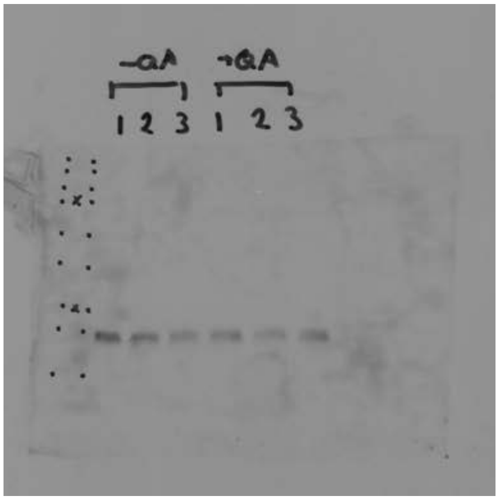

Figure 5 - A (cathepsin L)

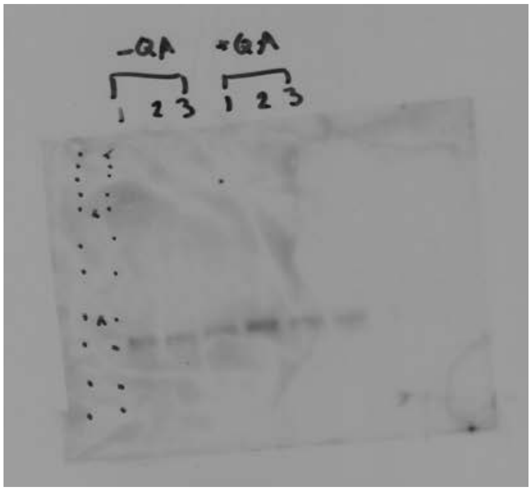

Figure 5 - A (p62/SQSTRM1)

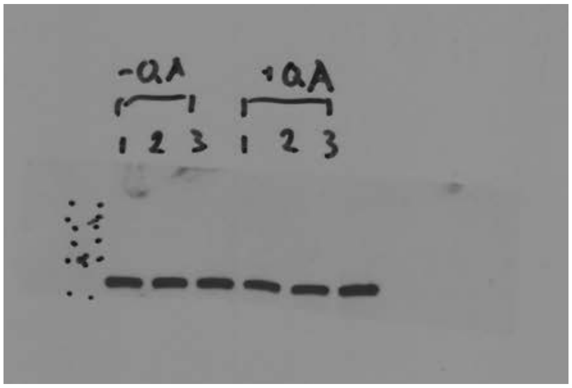

Figure 5 - A (LC3)

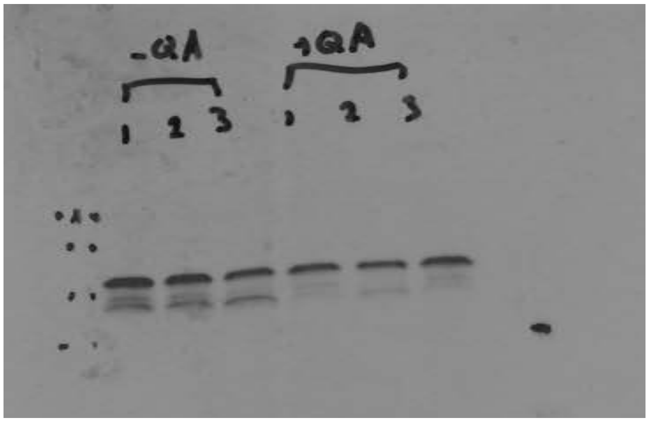

Figure 5 - A (Actin)

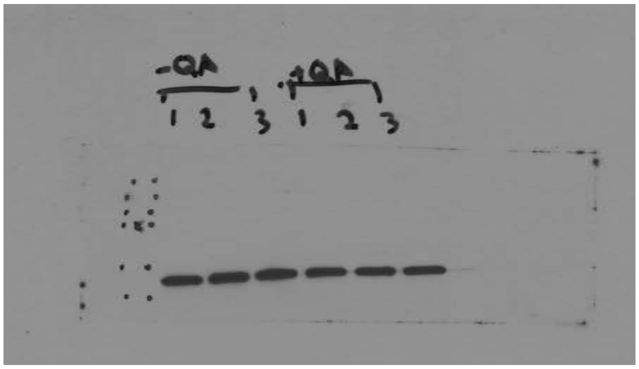

Supplement: Supplementary file 1 — Supplementary information [file 41598_2020_67505_MOESM1_ESM.pdf]
